# Quality Improvement to Improve the Referral Process to the Barnet Enhanced Support Team (BEST)

**DOI:** 10.1192/bjo.2026.11502

**Published:** 2026-06-30

**Authors:** Kieran Ferrier, Oliver Tran, Elizabeth Manning

**Affiliations:** North London NHS Foundation Trust, London, United Kingdom

## Abstract

**Aims:**

The Barnet Enhanced Support Team (BEST) are a multidisciplinary CAMHS service. BEST is a specialist Tier 3 CAMHS service to provide higher intensity support for children and young people (CYP) with complex mental health needs and their families. The aim is to improve the understanding of clinicians belonging to the Barnet CAMHS team of the BEST'sreferral criteria and what the service offers.

**Methods:**

1) A pre-intervention questionnaire was given to members of the Barnet CAMHS team. Questions included: whether they have heard of the BEST service, if they have referred to the BEST service in the past, if they know how to refer, how familiar they were with the referral criteria, how confident they were in knowing what services BEST offers, how easy they found the process, how it can be improved, what service they work with and their role.

2) An information poster was made outlining who the BEST service are, what the service does, and how to refer to the service. This poster was presented to members of the Barnet CAMHS team during the weekly multidisciplinary team (MDT) meeting.

3) A post-intervention questionnaire was given to members of the Barnet CAMHS team. Questions asked were the same as in the pre-intervention questionnaire in order to observe a change in response.

4) Data from the pre- and post-intervention questionnaires were analysed to identify changes and what further improvements can be made, thus providing insight for further quality improvement cycles in the future.

**Results:**

31 pre-intervention and 13 post-intervention questionnaires were collected. The number of people who know how to refer to the BEST service increased by 53.60%. The familiarity of people with the referral criteria (rated out of 10) increased by 54.97%. Confidence in knowing what the BEST service offers increased by 35.33%. Ease of the referral process (rated out of 10) increased by 48.57%. Respondents suggested that the referral process can be improved by attending the BEST MDT meetings in order to hear the outcome of the referral.

**Conclusion:**

The completion and presentation of the Barnet Enhanced Support Team (BEST) information poster produced an improvement in members of the Barnet CAMHS team's awareness of the service, including what the service offers and how to refer. Further improvements can be made through a collaborative approach with the respective teams, including by attending the BEST MDT meetings.

